# Congenital Bladder Diverticulum in Adults: A Case Report and Review of the Literature

**DOI:** 10.1155/2018/9748926

**Published:** 2018-01-14

**Authors:** Rawad Abou Zahr, Khalil Chalhoub, Farah Ollaik, Joe Nohra

**Affiliations:** ^1^Department of Urology, Saint George Hospital University Medical Center, University of Balamand, Beirut 1100 2807, Lebanon; ^2^Saint George Hospital University Medical Center, Faculty of Medicine, University of Balamand, Beirut 1100 2807, Lebanon

## Abstract

A 37-year-old male patient presented to the emergency department with fever, gross hematuria, and irritative lower urinary tract symptoms. Investigations revealed the presence of a large left bladder diverticulum superior and lateral to the left ureteral orifice without bladder outlet obstruction. Bladder diverticula in adults almost always present in the setting of bladder outlet obstruction. The finding of a congenital bladder diverticulum in an adult represents a rare clinical entity.

## 1. Introduction

Bladder diverticula are “hernias of the bladder mucosa through muscular fibers of the bladder wall” [1] resulting in a thin-walled structure that poorly empties during micturition. Bladder diverticula are either congenital or acquired. The incidence of congenital bladder diverticula is reported to be 1.7% [2] and peaks in children below 10 years of age [3]. 90% are located superolateral to the ureteral orifice in proximity to the ureterovesical junction [4]. Unlike the acquired adult form, in which outlet obstruction or neurogenic dysfunction is almost always present, congenital bladder diverticula result from hypoplasia of the muscular layer of the bladder wall [1]. Bladder diverticula usually present as incidental findings on imaging during investigation of lower urinary tract symptoms (LUTS), hematuria, or infection. We present the rare case of a congenital bladder diverticulum in an adult.

## 2. Case Presentation

A 37-year-old male, who is a 20 pack-year smoker and was previously healthy, presented to our emergency department with fever, gross hematuria, and irritative LUTS.

**Figure 1:**
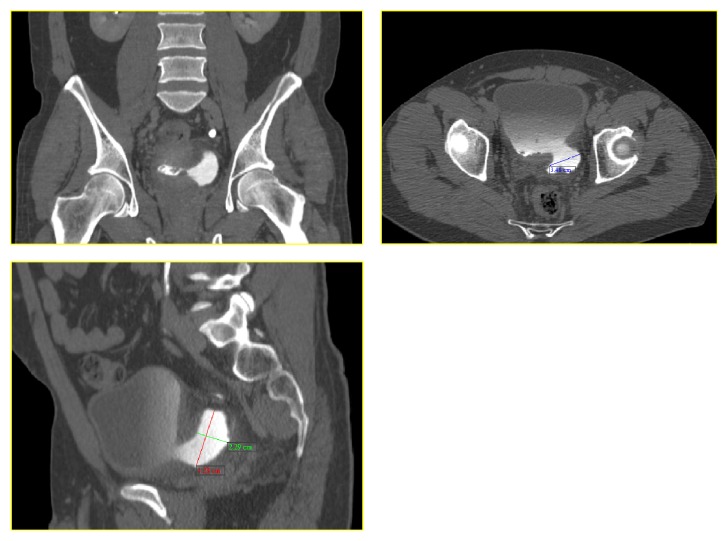
Coronal, sagittal, and axial cuts of bladder diverticulum on computed tomography.

CT scan of abdomen and pelvis revealed a well-distended bladder, with a significantly thickened and irregular wall measuring up to 1 cm with the presence of a 4 × 4 × 3 cm diverticulum at its left lower posterior aspect (Figure 1). An International Prostate Symptom Score (IPSS) done for evaluation of bladder outlet obstruction showed a score of 4 which falls in the mild category of symptoms.

**Figure 2:**
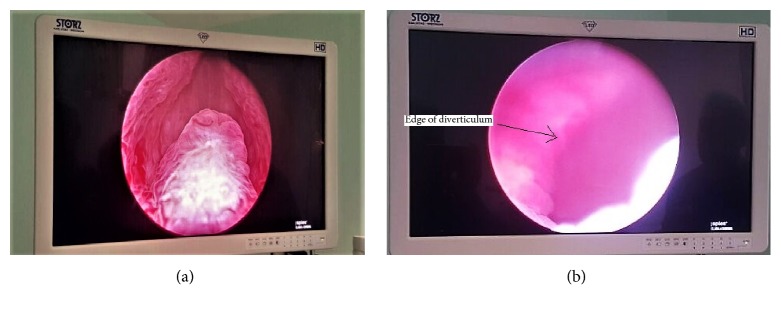
(a) Open prostatic fossa; (b) bladder diverticulum.

Cystoscopy revealed a large left bladder diverticulum superolateral to left ureteral orifice with an open prostatic fossa and no bladder trabeculations (Figure 2).

The patient noted an 80% improvement of his symptoms on intravenous antibiotics during his two-day hospitalization; he was discharged on oral cefixime for a total duration of 3 weeks. The merits of surgical excision versus surveillance were thoroughly discussed with the patient and he was counseled regarding periodic reassessment of symptoms with emphasis on follow-up ultrasound to evaluate bladder emptying. 

## 3. Discussion

Bladder diverticula are rare clinical entities in both the pediatric and adult populations. Their true incidence is not well established as most cases are asymptomatic or incidentally discovered on imaging. They were reported to be of approximately 1.7% according to a pediatric genitourinary database [2] and peak in children below 10 years of age [3]. They represent “hernias of the bladder mucosa through muscular fibers of the bladder wall” [1], resulting in a thin-walled structure in connection with the bladder lumen. Histological examination of resected surgical specimens supports the theory of “hypoplasia of the muscularis layer” as cause for herniation. The wall of the diverticulum is found to contain scattered, nonfunctional thin strands of smooth muscle which empty poorly during micturition [1]. This anomalous voiding leads to increased postvoid residue with paradoxical enlargement of the bladder diverticulum during micturition.

Bladder diverticula are classified as congenital or acquired. Congenital or primary bladder diverticula, which usually present during childhood [3] and occur in the absence of bladder outlet obstruction, are associated with smooth-walled bladder without trabeculations on cystoscopy [5] and bear no risk for malignancy as opposed to the secondary acquired type [6]. They are associated with congenital syndromes, namely, Ehlers-Danlos (type 9) syndrome, Menkes kinky hair syndrome, cutis laxa syndrome (Sotos), and Williams-Beuren syndrome [2, 4].

90% of primary congenital diverticula are in the vicinity of the ureterovesical junction [4]. A diverticulum located superolateral to the ureteral orifice without involving the trigone is referred to as “Hutch diverticulum” and it usually occurs in the setting of a neuropathic bladder and vesicoureteral reflux [5]. The most common presentation is urinary tract infections secondary to urinary stasis in the diverticulum. Other presentations may include acute urinary retention, bladder stones, enuresis, and possible bladder obstruction if the diverticulum enlarges and obstructs the bladder neck distally [1, 7].

Observation is usually advised if the patient is asymptomatic. Surgical management via diverticulectomy is warranted whenever the patient suffers from recurrent UTIs, bladder stones, complicated vesicoureteral reflux, voiding dysfunction, and urinary retention [8].

The surgical options include open diverticulectomy (intra- or extravesical) which may be beneficial in case of concomitant prostatic enlargement allowing simultaneous treatment of both entities. The extravesical approach is reserved for patients with large diverticula associated with peridiverticular adhesions or inflammation [8].

Laparoscopic approach may also be performed offering the advantages of minimally invasive surgery. Endoscopic transurethral incision of diverticular neck is reserved for nonsurgical candidates [9].

*Adult bladder diverticulum in the absence of bladder outlet obstruction represents a rare clinical finding. We report this case due to its unusual adult presentation*.

## 4. Conclusion

Bladder diverticula in adults are mostly secondary to bladder outlet obstruction; we report a rare case of congenital bladder diverticulum in an adult. Most congenital bladder diverticula are asymptomatic and are managed conservatively, unless patient develops complications due to voiding dysfunction, which then warrants surgical intervention.

## References

[1] Garat J. M., Angerri O., Caffaratti J., Moscatiello P., Villavicencio H. (2007). Primary Congenital Bladder Diverticula in Children. *Urology*.

[2] Blane C. E., Zerin J. M., Bloom D. A. (1994). Bladder diverticula in children. *Radiology*.

[3] Boechat M. I., Lebowitz R. L. (1978). Diverticula of the bladder in children. *Pediatric Radiology*.

[4] Psutka S. P., Cendron M. (2013). Bladder diverticula in children. *Journal of Pediatric Urology*.

[5] Hutch J. A. (1952). Vesico-ureteral reflux in the paraplegic: cause and correction. *The Journal of Urology*.

[6] Alexander R. E., Kum J. B., Idrees M. (2012). Bladder diverticulum: Clinicopathologic spectrum in pediatric patients. *Pediatric and Developmental Pathology*.

[7] Oge O., Gemalmaz H., Ozeren B. (2002). Acute urinary retention in a child caused by a congenital bladder diverticulum. *Journal of Pediatric Surgery*.

[8] Evangelidis A., Castle E. P., Ostlie D. J., Snyder C. L., Gatti J. M., Murphy J. P. (2005). Surgical management of primary bladder diverticula in children. *Journal of Pediatric Surgery*.

[9] Silay M. S., Koh C. J. (2015). Management of the Bladder and Calyceal Diverticulum: Options in the Age of Minimally Invasive Surgery. *Urologic Clinics of North America*.

